# Reliability of self-reported questionnaire for epidemiological investigation of *Helicobacter pylori* eradication in a population-based cohort study

**DOI:** 10.1038/s41598-021-95124-1

**Published:** 2021-08-02

**Authors:** Yu Sasaki, Yasuhiko Abe, Masakuni Shoji, Naoko Mizumoto, Hiroaki Takeda, Harufumi Oizumi, Takao Yaoita, Norie Sawada, Kazumasa Yamagishi, Eiko Saito, Masafumi Watanabe, Kenichi Ishizawa, Tsuneo Konta, Takamasa Kayama, Shoichiro Tsugane, Yoshiyuki Ueno, Manami Inoue

**Affiliations:** 1 Department of Gastroenterology, Faculty of Medicine, Yamagata University, 2-2-2 Iida-Nishi, Yamagata, 990-9585 Japan; 2 Division of Endoscopy, Yamagata University Hospital, 2-2-2 Iida-Nishi, Yamagata, 990-9585 Japan; 3 Department of Gastroenterology, Yamagata Prefectural Central Hospital, 1800 Aoyagi, Yamagata, 990-2292 Japan; 4 Gastroenterology and Internal Medicine, Oizumi Medical Clinic, 5-21-15 Shironishimachi, Yamagata, 990-0832 Japan; 5 Yaoita Clinic, 1-2-29 Tokamachi, Yamagata, 990-0031 Japan; 6 Division of Cohort Research, Center for Public Health Sciences, National Cancer Center, 5-1-1 Tsukiji, Chuo-ku, Tokyo, 104-0045 Japan; 7 Department of Public Health Medicine, Faculty of Medicine, and Health Services Research and Development Center, University of Tsukuba, 1-1-1 Tennodai, Tsukuba, 305-8575 Japan; 8 Division of Cancer Statistics Integration, Center for Cancer Control and Information Services, National Cancer Center, 5-1-1 Tsukiji, Chuo-ku, Tokyo, 104-0045 Japan; 9 Insitute for Promotion of Medical Science Research, Yamagata University, 2-2-2 Iida-Nishi, Yamagata, 990-9585 Japan; 10 National Institute of Health and Nutrition, National Institutes of Biomedical Innovation, Health and Nutrition, 1-23-1 Toyama, Shinjuku-ku, Tokyo, 162-8636 Japan; 11 Division of Prevention, Center for Public Health Sciences, National Cancer Center, 5-1-1 Tsukiji, Chuo-ku, Tokyo, 104-0045 Japan

**Keywords:** Gastrointestinal diseases, Epidemiology

## Abstract

General population-based cohort studies provide solid evidence on mass *Helicobacter pylori* (HP) eradication effects. Self-reported questionnaires are occasionally used in such studies to ascertain the HP eradication history. However, reports on the reliability of these questionnaires are lacking. This general population-based cohort study included 899 individuals with HP infection at the baseline survey who were reported to have eradicated it at the 5-year follow-up survey. Of these, the medical records of 280 patients were available for investigation, and the HP eradication status of 93 individuals was ascertained. Their medical records were reviewed, and the reliability of the self-reported questionnaire responses was assessed. Of the 91 individuals who successfully eradicated HP based on the medical records, 90 (98.9%) answered the self-reported questionnaire correctly, with an unweighted kappa value of 0.661 (*p* < 0.001). The difference between the self-reported and medical records age at eradication was within a 1-year range in most participants (86.8%). Similarly, the HP eradication procedure and the outcomes were reasonably matched. In conclusion, the responses to the self-reported HP eradication questionnaire were almost consistent with the medical records. Thus, HP eradication history assessment by a self-reported questionnaire is reliable for an epidemiological study in the general population.

## Introduction

*Helicobacter pylori* (HP) is a gram-negative, microaerophilic bacterium that infects the gastric epithelium. It is estimated that more than half of the world’s population is infected with HP^1^, making it the most widespread infection worldwide. Overall, HP infection shows a progressive decline in prevalence; however, in some Middle East countries, the prevalence has remained relatively stable^2^. The prevalence of HP in Japan has been rapidly decreasing partly due to the increase in the number of patients cured of HP infection since 2013, when the health insurance policy started covering HP gastritis^3^.

It is well known that HP infection contributes to the development of chronic gastritis, peptic ulcer disease, and gastric cancer^4,5^. The International Agency for Research on Cancer (IARC), a subsidiary of the World Health Organization, categorized HP in 1994 as a group 1 carcinogen for gastric cancer^6^, the fifth most frequently diagnosed cancer, and the third most common cause of cancer-related death worldwide^7^. The IARC reported that 2.2 million new cancer cases were attributable to infections in 2018, and HP was responsible for 810,000 of these cases^8^. The age-standardized HP infection incidence rate was estimated at 8.7 cases per 100,000 person-years, making HP the most important infectious cause of cancer worldwide^8^.

There is significant interest in mass HP eradication for gastric cancer prevention. The Kyoto global consensus report recommended that all individuals with HP infection should receive eradication therapy to prevent gastric cancer^9^. However, eradication benefits for baseline gastric cancer incidence vary across regions and populations^10^. A recent Cochrane Database of Systematic Reviews study reported moderate evidence that investigating and eradicating HP reduced the incidence of and mortality from gastric cancer in asymptomatically infected Asian individuals^11^. Similarly, there was minimal evidence of benefits in terms of all-cause mortality and adverse events.

Therefore, several epidemiological studies have evaluated the effect of HP eradication on gastric cancer; however, its beneficial or adverse effects remain uncertain, especially in the general population^12^. Therefore, large-scale cohort studies are necessary to clarify the effects of HP eradication. Such analysis of the outcomes is extremely important in Japan, the only country in the world that promotes HP eradication therapy to all patients with HP infection^13^. Indeed, self-reported questionnaires should be used to obtain the HP eradication history in a large-scale general population-based cohort study. It is necessary to collate the self-reported results with the medical records to ensure their accuracy; however, this becomes less easy as the survey scale increases. Furthermore, there are no reports clarifying the reliability of such questionnaires on HP eradication history. Therefore, this study aimed to examine the reliability of HP eradication history and its therapeutic outcomes, obtained through a self-reported questionnaire in a general population cohort.

## Results

### Consistency of HP eradication status between the medical records and self-reported responses

HP eradication status was confirmed in the medical records of 92 individuals, including 32.8% of the 280 individuals whose medical records were investigated (Table 1). Of 91 patients with successful HP eradication based on the medical records, 90 (97.8%) reported successful eradication in the self-reported questionnaire. Only one individual was confirmed on both the medical records and the self-reported questionnaire to have failed to eradicate HP. The unweighted kappa value for the HP eradication results between the self-reported response and the medical records was 0.661 (95% confidence interval [CI], 0.041–1.000, *p* < 0.001). The HP was eradicated by the second-line treatment in the individual who provided an incorrect response.

**Table 1:** Comparison of the HP eradication status between the medical records and self-reported responses.

Medical records *n* = 280		Self-reported responses *n* = 280	Concordance rate (%)
Results of HP eradication treatment	*n* (%)	Success/failure, *n* (%)	
**All**			
Success	91 (32.5)	90 (98.9)/1 (1.0)	98.9
Failure	1 (0.3)	0 (0)/1 (100)	100
Unknown^a^	1 (0.3)	1 (100)/0 (0)	–
No record^b^	187 (66.7)	178 (95.1)/9 (4.8)	–
**First-line HP eradication**			
Success	72 (77.4^c^)	72 (100)/0 (0)	100
Failure	21 (22.5^c^)	–	–
**Second-line HP eradication**			
Success	19 (90.4^d^)	18 (94.7)/1 (5.2)	94.7
Failure	0 (0^d^)	–	–
Unknown^a^	1 (4.8^d^)	1 (100)/ 0 (0)	–
Refused	1 (4.8^d^)	0 (0)/ 1 (100)	–

Values are expressed as number (percentage). No participant received third-line treatment.

HP, *Helicobacter pylori.*

^a^This individual confirmed receiving a second-line HP eradication treatment; however, no description of its outcome was found.

^b^The medical records of these participants contained no description regarding their HP eradication treatment at the participating institutions.

^c^The percentage was calculated by dividing the number of successes or failures by the number of participants with medical records (*n* = 93).

^d^The percentage was calculated by dividing the number of individuals by the number of the first-line treatment failures (*n* = 21).

### Consistency of age at HP eradication between the medical records and self-reported responses

**Figure 1:**
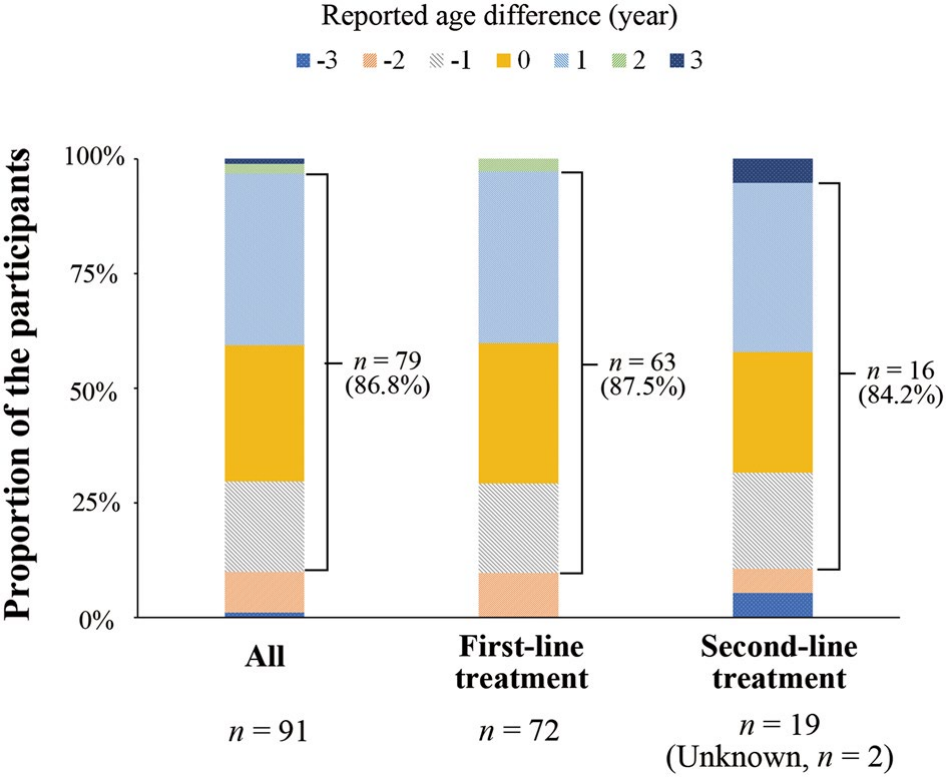
Differences in *Helicobacter pylori* (HP) eradication age between the medical records and self-reported responses. The eradication age differences between the medical records and self-reported responses are indicated by the following colors: blue, minus 3 years; orange, minus 2 years; gray, minus 1 year; yellow, no difference; sky blue, plus 1 year; green, plus 2 years; navy blue, plus 3 years.

The self-reported age at HP eradication exactly matched the medical records in 27 individuals (29.7%), comprising 22 (30.6%) and five (26.3%) individuals with HP eradicated by the first- and second-line treatments, respectively (Fig. 1). The unweighted kappa values for the age at eradication between the self-reported response and the medical records were 0.177 (95% CI, 0.080–0.274, *p* < 0.001) for all, 0.184 (95% CI, 0.075–0.292, *p* < 0.001) in those with HP eradicated by the first-line treatment, and 0.130 (95% CI, − 0.065 to 0.326, *p* = 0.129) in those with HP eradicated by the second-line treatment. Only a few individuals had a complete match between the self-reported age at HP eradication and the medical records; however, the difference in the age of eradication for most individuals was within 1 year [79 individuals (86.8%) overall, 63 (87.5%) with HP eradicated by the first-line treatment, and 16 (84.2%) with HP eradicated by the second-line treatment; Fig. 1].

### Impact of individual characteristics on the differences in reported eradication age between the medical records and self-reported responses

No differences were observed in sex, birth age, smoking or alcohol drinking habit, exercise habit, educational background, and median height, weight, and body mass index between individuals whose self-reported age of eradication differed within 1 year from the medical records and those whose age of eradication differed by 2 years or more (Table 2). Individuals with a history of health checkups for gastric cancer were significantly more likely to have a difference of 2 years or more between the self-reported and medical records eradication age (Table 2).

**Table 2:** Factors associated with the differences in HP eradication age between the medical record and self-reported responses.

Baseline individual characteristics	Difference (year)		*p* value
	0 ± 1, *n* = 79	≥ 2, *n* = 12	
Male/female	27 (34.1)/52 (65.8)	3 (25.0)/9 (75.0)	0.52
Age, years	64 (61–68)	62 (53.2–66.2)	0.16
Height, cm	158.9 (153.3–163.6)	156.1 (152.8–164.1)	0.50
Weight, kg	54.2 (50.4–65.5)	52.3 (49.1–66.9)	0.69
BMI^a^, kg/m^2^	22.1 (20.5–24.0)	21.7 (20.1–23.6)	0.77
**Smoking habits**			0.82
Never	54 (68.3)	9 (75.0)	0.64^e^
In the past	15 (18.9)	2 (16.6)	
Current	5 (6.3)	0 (0)	
No answer	5 (6.3)	1 (8.3)	
**Alcohol drinking habits**			0.67
Daily drinking^b^	15 (18.9)	2 (16.6)	0.70^e^
Less than 3 days a week	12 (15.1)	1 (8.3)	
Rarely or none^c^	47 (59.4)	9 (75.0)	
No answer	5 (6.3)	0 (0)	
**Exercise habit**^d^			0.37
Yes/ No	19 (24.0)/3 (3.8)	5 (41.6)/0 (0)	0.38^e^
No answer	57 (72.1)	7 (58.3)	
**GI health checkup history**			
For gastric cancer	50 (63.3)	12 (100)	< 0.01
For colorectal cancer	7 (8.8)	1 (8.3)	1.00
Either/ both	43 (54.4)/7 (8.8)	11 (91.6)/1 (8.3)	0.03
**Educational background**			0.72
Junior high school graduate	6 (7.5)	0 (0)	0.93^e^
High/vocational school graduate	63 (79.7)	10 (83.3)	
University/college graduate	9 (11.3)	2 (16.6)	
No answer	1 (1.2)	0 (0)	

Values are expressed as number (percentage) or median (interquartile range).

The groups were compared by the Wilcoxon rank-sum test or Chi-squared test.

*BMI* body mass index, *GI* gastrointestinal.

^a^BMI was calculated as weight in kilograms divided by height in meters squared.

^b^Daily alcohol drinking was defined as drinking at least 3 days per week.

^c^Rarely or none was defined as less than 2 days per month or none at all.

^d^The self-reported questionnaire asked the individuals to evaluate their exercise habits using the following sentence: have you exercised to sweat lightly for 30 min a day at least two times a week for more than 1 year?

^e^*p* values were calculated after excluding those who did not answer the question.

### HP eradication therapy

HP was eradicated in almost all individuals by a proton pump inhibitor (PPI)- or potassium-competitive acid blocker (p-CAB)-based triple therapy administered for 7 days (Table 3). The first-line and second-line eradication rates in this study were 77.4 and 95.0%, respectively. HP eradication rate was significantly higher with p-CAB-based therapy than with PPI-based therapy (*p* = 0.04). HP eradication was determined by ^13^C-urea breath tests using a cut-off value of 2.5‰ and/or a negative rapid urease test. The incidence of adverse events was 4.3% and 5.0% during the first-line and second-line eradication, respectively.

**Table 3:** *Helicobacter pylori* (HP) eradication therapy in the individuals whose medical records were available for review.

Regimens^a^	Duration	Success *n* (%)	Failure *n* (%)	Eradication confirmation tests^b^, *n* (%)		Adverse effects
				UBT	RUT	
First-line treatment, *n* = 93		72 (77.4)	21 (22.5)	89 (95.6)	4 (4.3)	4 (4.3)
EPZ + AMPC + CM	7 days	3 (60.0)	2 (40.0)	5 (100)	0 (0)	None reported
LPZ + AMPC + CM	7 days	20 (64.5)	11 (35.4)	30 (96.7)	1 (3.2)	1 diarrhea + skin rash + nausea, 1 dizziness, 1 diarrhea
RPZ + AMPC + CM	7 days	30 (81.0)	7 (18.9)	34 (91.8)	3 (8.1)	1 altered taste
VPZ + AMPC + CM	7 days	15 (100)	0 (0)	14 (100)	0 (0)	None reported
Unknown, *n* = 5	–	4 (80.0)	1 (20.0)	5 (100)	0 (0)	None reported
Second-line treatment, *n* = 20		19 (95.0)	0 (0)	19 (95.0)	0 (0)	1 (5.0)
EPZ + AMPC + MNZ	7 days	3 (100)	0 (0)	3 (100)	0 (0)	None reported
LPZ + AMPC + MNZ	7 days	5 (100)	0 (0)	5 (100)	0 (0)	None reported
OPZ + AMPC + MNZ	7 days	1 (100)	0 (0)	1 (100)	0 (0)	1 diarrhea
RPZ + AMPC + MNZ	7 days	10 (100)	0 (0)	10 (100)	0 (0)	None reported
Unknown, *n* = 1	–	The result was not reported^c^				

Values are expressed as number (percentage).

^a^Proton pump inhibitor- or potassium-competitive acid blocker-based triple therapy was used: twice daily 20 mg esomeprazole (EPZ), 30 mg lansoprazole (LPZ), 20 mg omeprazole (OPZ), 20 mg rabeprazole (RPZ), or 20 mg vonoprazan (VPZ). All combined with twice-daily administration of 750 mg amoxicillin (AMPC) and 200 mg clarithromycin (CM) or 250 mg metronidazole (MNZ).

^b^HP eradication was determined by ^13^C-urea breath tests (UBT) with a cut-off value of 2.5‰ and/or negative rapid urease test (RUT).

^c^One individual confirmed receiving a second-line HP eradication treatment; however, no description of its outcomes was found.

## Discussion

This is the first study demonstrating an optimal internal consistency in HP eradication history results between self-reported questionnaires and the medical records. Mostly, differences in the reported age of eradication remained within 1 year. These results suggest that the studied self-reported questionnaire for HP eradication history is valid and a suitable tool for HP eradication history assessment in the general population.

Recent large-scale cohort studies evaluated the effect of HP eradication treatment on the prevention of gastric cancer by assessing the HP eradication history with various survey methods^12,14,15^. The HP eradication history was collected from the unified electronic medical records of all Veterans Health Administration (VHA) facilities for a retrospective cohort study with data of 371,813 patients in the VHA^15^. Another hospital-based study^12^ investigated the HP eradication history based on a self-reported questionnaire but did not report its reliability. In hospital-based cohort studies, it is generally considered that the HP eradication history should rely on a medical interview or self-reported questionnaire. Furthermore, the Swedish drug registry was used to obtain information on HP eradication treatment in a population-based, nationwide (n = 95,176) cohort study in Sweden^14^; however, eradication success was not confirmed. A survey method that strikes a balance between the accuracy and feasibility of the eradication history acquisition is required to more precisely estimate HP eradication effectiveness in large-scale cohort studies. A self-reported questionnaire might be a proper tool for examining the effect of HP eradication with valid confidence in general population cohort studies.

A history of health checkups for gastric cancer was more observed among the individuals whose self-reported age of eradication differed by more than 2 years from the medical records than among those with a difference of within 1 year. It is assumed that individuals with a history of such health checkups are more health-conscious and participate in various health-related activities. This could have contributed to the confusion in recalling the age at which HP was eradicated. Therefore, the extent of differences in the self-reported age at HP eradication might vary between hospital- and general population-based cohort studies. In addition, since individuals had to report the age by a number, the reported age depended on how it was rounded. Similarly, this could have contributed to the age difference from the medical records.

The medical records confirmed the actual eradication regimen and the method for efficacy evaluation in our study. Almost all individuals received PPIs- or p-CAB-based triple therapy for HP eradication following the guidelines for the management of HP infection in Japan^16^. Eradication efficacy was primarily determined by ^13^C-urea breath tests. The overall eradication rate was 77% for first-line eradication and 95% for second-line eradication, similar to those observed in previous reports^17^. Supposedly, the current decline in the first-line eradication rate in Japan is due to the impact of the high clarithromycin (CM) resistance rate of approximately 30%. In fact, metronidazole-based eradication therapy has been proven to be more effective than CM-based eradication therapy in a randomized trial conducted in Japan^18^. Thus, the low eradication rate observed for first-line eradication compared to second-line eradication in this study may be due to the influence of CM resistance. As previously reported^19,20^, the first-line HP eradication rate was higher with p-CAB-based triple therapy than with PPI-based triple therapy. p-CAB allows rapid, profound, and sustained suppression of gastric acid secretion^21^, which is an important factor for increasing the antibiotics sensitivity of HP^22^. The cytochrome P450 2C19 (CYP2C19) plays a role in the effect of PPIs on acid secretion, and a decrease in eradication rate has been reported in CYP2C19 extensive metabolizers^23^. In contrast, the effects of p-CAB and the eradication rate on p-CAB-based triple therapy are not influenced by CYP2C19 polymorphism^19^. Furthermore, a recent meta-analysis demonstrated that p-CAB-based therapy is superior to PPI-based therapy to eradicate CM-resistant HP strains^24^. These factors may explain the high eradication rate of p-CAB-based therapy compared to PPI-based therapy observed in this study.

This study had several limitations. It was feasible to collate the self-reported questionnaire results with the medical records for only a few individuals who visited two hospitals and two clinics in a single prefecture. This could affect the generalizability of the results to other populations. Therefore, further validation studies with a larger number of participants are required. The self-reported questionnaire reliability might be affected by differences in ethnicity, education, background, HP infection rate, and gastric cancer incidence rate. Therefore, the questionnaire should be validated in various other settings, and the content of the questionnaire and the questionnaire design should be clarified. To verify the effects of HP eradication interventions, it is important to confirm the success or failure of HP eradication and whether persistently infected patients truly do not have a history of HP eradication. However, a medical record survey is required at all hospitals or clinics visited by the individuals to verify no history of HP eradication, which could not be conducted in this study. The reliability of the self-reported questionnaire in these individuals may differ from the individuals in this study. Hence, additional validation, including individuals who reported no HP eradication, is required. The adverse effects of HP eradication therapy were reviewed retrospectively from a medical record survey. Therefore, the actual incidence of adverse effects may have been underestimated.

In conclusion, the HP eradication results on the self-reported questionnaire in this study were almost consistent with the information in the medical records. Although a complete agreement on the HP eradication age was not achieved, the differences were mostly within 1 year. This difference may have been influenced by the history of health check-ups for gastric cancer. Therefore, HP eradication history assessment by a self-reported questionnaire could be considered reliable and valid in epidemiological studies in the general population.

## Methods

### Study population

**Figure 2:**
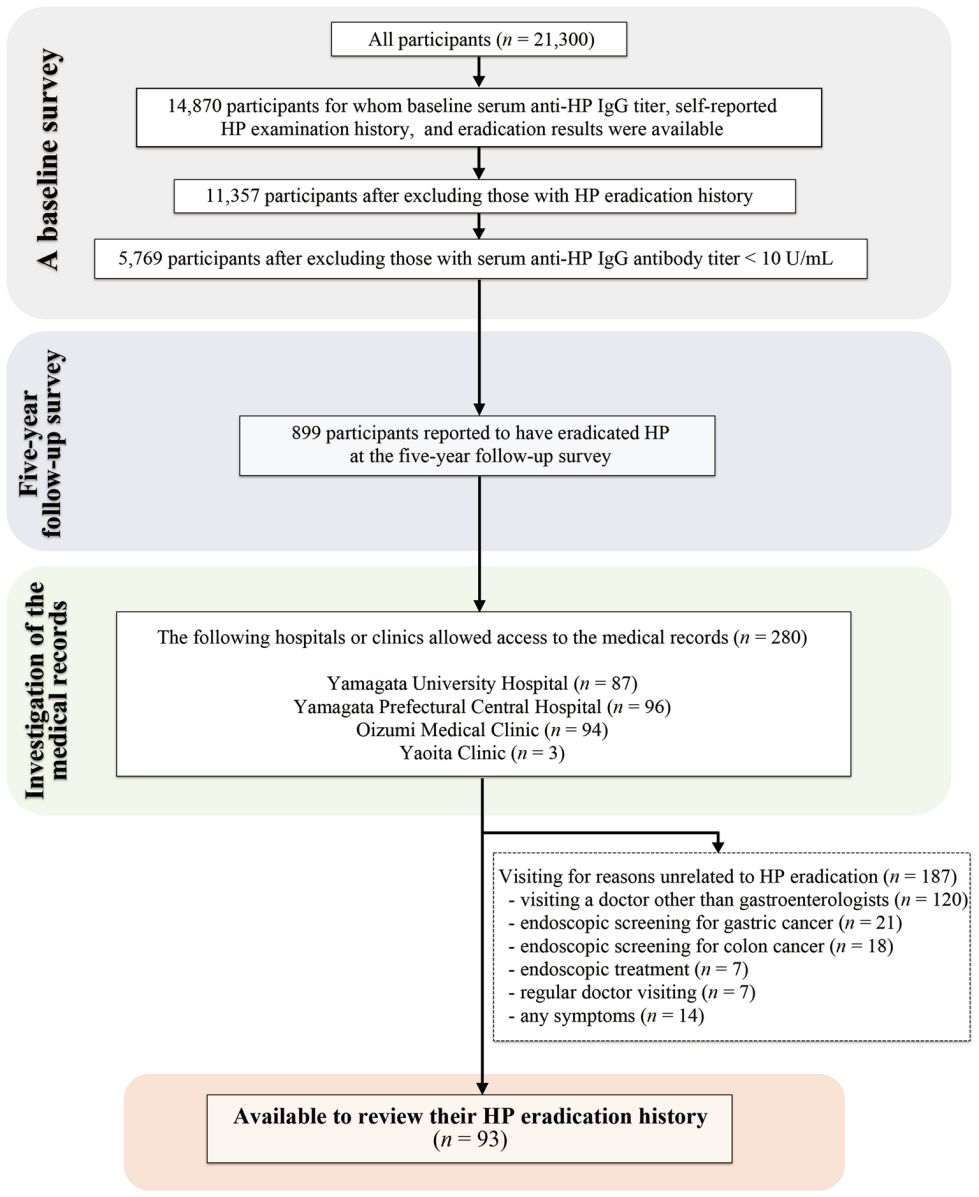
Flowchart showing the study participant selection process.

**Figure 3:**
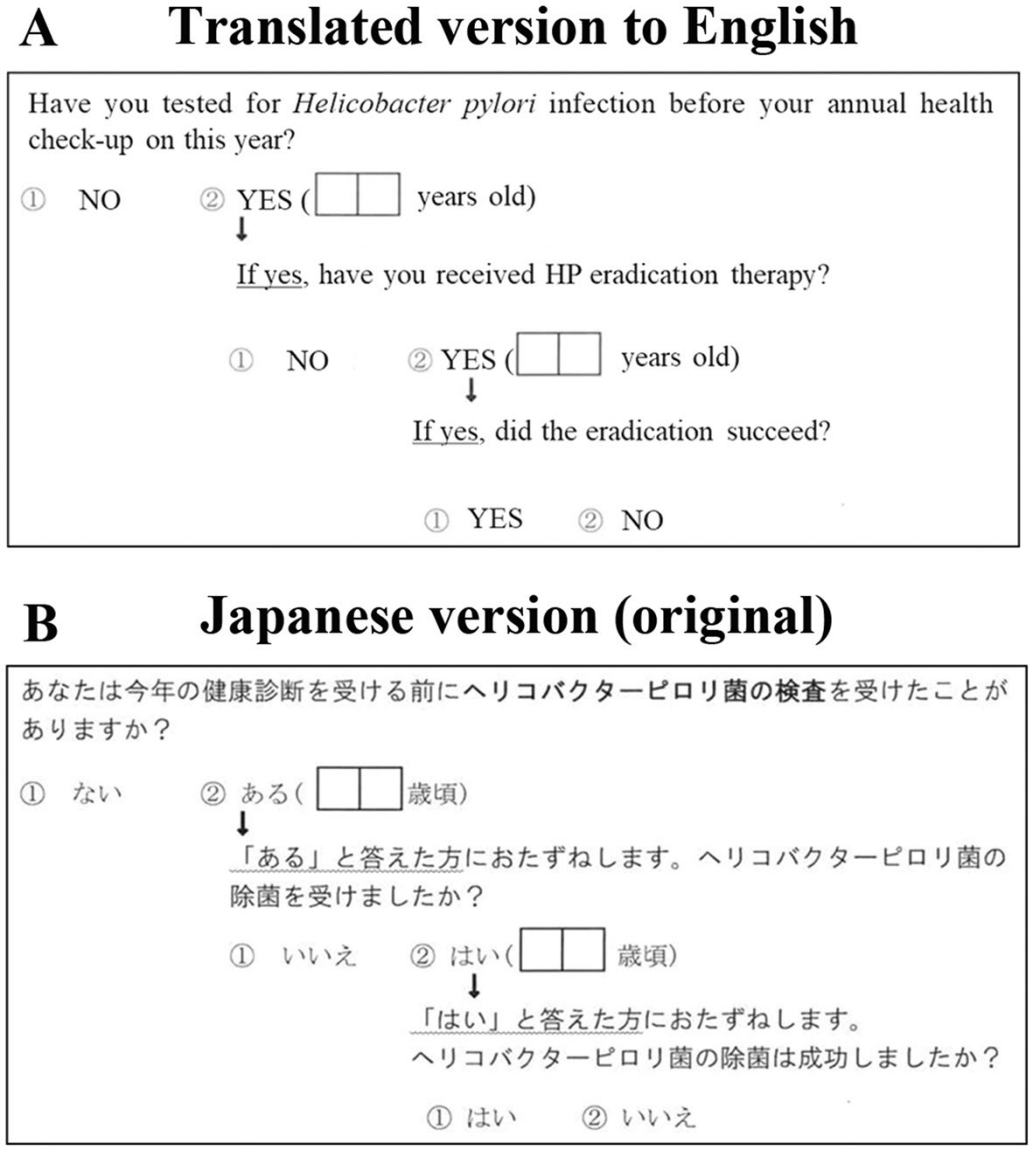
A self-reported questionnaire about HP eradication history in our population-based cohort. The upper panel is the English translation of the original (**A**), and the lower panel shows the original Japanese version (**B**).

Participants in this study were enrolled from a general population-based cohort study, named the Yamagata study, that aims to identify novel therapeutic targets through the elucidation of risk factors, such as environmental factors and genetic background and their underlying pathogeneses in the development of lung, stomach, colorectal, liver, and breast cancers, as well as lifestyle-related diseases, such as stroke, acute myocardial infarction, hypertension, renal failure, and diabetes^25^. Participants in the Yamagata study underwent community-based annual health check-ups in seven cities (Yamagata-city, Sakata-city, Sagae-city, Yonezawa-city, Kaminoyama-city, Tendo-city, and Higashine-city) in Yamagata Prefecture, Japan, from 2009 to 2015. All participants agreed to be included in this cohort study (*n* = 21,300). The participants were investigated at baseline by a self-reported questionnaire and data collected from their health check-up results. All participants were scheduled to undergo a 5-year follow-up, and their self-reported questionnaires and health check-up results were collected at the follow-up survey. The selection flowchart for inclusion in this study is shown in Fig. 2. The HP infection status based on serum anti-HP immunoglobulin G (IgG) antibody titer, self-reported HP examination history, and the eradication results at the baseline survey (Fig. 3) were available for 14,870 individuals. Serum anti-HP IgG antibody titers were ≥ 10 U/mL in 5769 of 11,357 individuals with no history of HP eradication at the baseline survey. Of these, 899 individuals were reported to have eradicated HP at the 5-year follow-up survey. The health insurance database was searched for participant hospital visits, and the medical records were examined to evaluate the validity of the self-reported questionnaire regarding HP eradication history. For practical reasons, four medical institutions, two major hospitals, and two gastroenterology clinics in Yamagata City were available to validate the questionnaire information. These included the Yamagata University Hospital (*n* = 87), Yamagata Prefectural Central Hospital (*n* = 96), Oizumi Medical Clinic (*n* = 94), and Yaoita Clinic (*n* = 3). Among the 280 investigated medical records, 93 individuals could be evaluated for their HP eradication history. The other 187 individuals had visited the four institutions for reasons unrelated to HP eradication, such as visiting a doctor other than a gastroenterologist (*n* = 120), endoscopic screening for gastric (*n* = 21) or colorectal (*n* = 18) cancer, endoscopic treatment (*n* = 7), regular doctor visit (*n* = 7), and due to other unrelated symptoms (*n* = 14).

Of the 5769 HP-positive individuals, 4870 included those whose self-reported questionnaires were yet to be confirmed at the time of this study (*n* = 3587), those who reported having no HP eradication (*n* = 710), those for whom no clear age of eradication was mentioned (*n* = 250), and those who did not provide any description of their HP eradication history (*n* = 323).

This study was approved by the Ethics Review Committees of Yamagata University Faculty of Medicine (#2019-175), Yamagata Prefectural Central Hospital (October 2019, #103), Oizumi Medical Clinic, and Yaoita Clinic, and was conducted in accordance with the Declaration of Helsinki. Written informed consent was obtained from all participants at enrollment in this cohort study.

### Medical records investigation

We (YS, YA, MS, NM, HT, HO, and TY), all board-certified gastroenterologists of the Japanese Society of Gastroenterology, together with some board-certified HP infection physicians of the Japanese Society for *Helicobacter* Research (YS, YA, and HO), visited the medical institutions and reviewed the medical records regarding HP eradication treatment, including treatment regimens, outcomes, testing for eradication confirmation, adverse effects, the date of eradication, and explanation of the results.

### Evaluation of serum anti-HP IgG antibody titer

Serum anti-HP IgG antibody titers were measured by enzyme-linked immunosorbent assay (ELISA) using the E-Plate Eiken or E-Plate II Eiken (Eiken Chemical Co., Ltd., Tokyo, Japan), following the manufacturer's instructions. Values greater than 10 U/mL were considered to indicate HP infection.

### Statistical analysis

Data are presented as numbers (percentages) or medians (interquartile ranges). Continuous or categorical variables were compared by the two-tailed Wilcoxon rank-sum test or chi-squared test, respectively. The concordance rate between the self-reported questionnaire and the medical records was assessed by the unweighted or weighted kappa coefficient. All statistical analyses were performed using JMP, Version 14.3.0 (SAS Institute Inc., Cary, NC, USA). Values of *p* < 0.05 were considered statistically significant.

## Data Availability

All relevant original data are available from the corresponding author upon reasonable request.
